# Supplementary material for: Awareness, knowledge, and attitude towards basic life support among healthcare professional students in Bangladesh
Source: PLOS Glob Public Health. 2025 Sep 2;5(9):e0005126. doi: 10.1371/journal.pgph.0005126 (PMC12404403; doi:10.1371/journal.pgph.0005126)
Supplement: S1 Text — (DOCX) [file pgph.0005126.s002.docx]

Awareness, knowledge and attitude towards basic life support among healthcare professional students in Bangladesh

| Q. N | Question | Answer | |
| --- | --- | --- | --- |
| **Section A Socio-demographic question** | | | |
| 1 | Gender | Male |  |
|  |  | Female |  |
| 2 | Department | Physiotherapy |  |
|  |  | Nursing |  |
|  |  | MBBS |  |
| 3 | Attained BLS training | Yes |  |
|  |  | No |  |
| 4 | Attained ALS training | Yes |  |
|  |  | No |  |
| **Section B Awareness and attitude towards BLS** | | | |
| 5 | What is BLS | Best life support |  |
|  |  | Best life service |  |
|  |  | Basic life support |  |
|  |  | Best level service |  |
| 6 | Do you think everyone should have the knowledge regarding BLS | Yes |  |
|  |  | No |  |
| 7 | Do you recommend BLS in your academic curriculum | Yes |  |
|  |  | No |  |
| **Section C basic knowledge of BLS** | | | |
| 8 | BLS can be performed at | Only hospital setup |  |
|  |  | Both inside and outside the hospital |  |
| 9 | Do you observed BLS being performed | Yes |  |
|  |  | No |  |
| 10 | If you don’t want to give mouth to mouth CPR, what can be done | Place your hand in front of the mouth and breath |  |
|  |  | Place a cloth in front of the mouth and breath |  |
|  |  | Blow air to the mouth |  |
| 11 | What do you do first when you get an unconscious person | Observe victims’ responsiveness |  |
|  |  | Call on emergency number |  |
|  |  | CAB |  |
|  |  | Above all 3 |  |
|  |  | Leave the place for safety |  |
| 12 | Please indicate the dialing number for help in case of medical emergency |  | |
| 13 | How do you know when to start compression | Victim has no detectable pulse |  |
|  |  | When victim is unconscious |  |
|  |  | Victim has a definite detectable pulse but not breathing |  |
| 14 | Should start CPR within ……seconds of recognition of cardiac arrest. | 5 |  |
|  |  | 10 |  |
|  |  | 15 |  |
|  |  | 20 |  |
| 15 | How do you check pulse of an unconscious person? | Check carotid artery on your side of the victim’s neck |  |
|  |  | Check carotid artery on your opposite side of the victim’s neck |  |
|  |  | Check brachial pulse |  |
| **Section C Knowledge of individual component of BLS** | | | |
| 16 | What is the compression rate of external cardiac massage per-minutes during BLS | 100-120 |  |
|  |  | 60-80 |  |
|  |  | 80-100 |  |
|  |  | 120-140 |  |
| 17 | What is the ratio of cardiac compression to breaths delivery during BLS | 30:1 |  |
|  |  | 30:2 |  |
|  |  | 30:3 |  |
|  |  | 30:4 |  |
| 18 | What is the rescue breathing rate per minute | 8 |  |
|  |  | 10 |  |
|  |  | 12 |  |
| 19 | Location of chest compression | Over the sternum |  |
|  |  | Over the left lower half of the sternum |  |
|  |  | Over the right lower half of the sternum |  |
| 20 | Sequence to be followed while performing BLS | ABC |  |
|  |  | CAB |  |
|  |  | ABCD |  |
| 21 | Depth of chest compression in adults during CPR | 2 to 2.4 inch |  |
|  |  | 2 to 2.4 cm |  |
| 22 | Performed BLS by self | Yes |  |
|  |  | No |  |
| 23 | For how long the pulse should be checked | Not more than 10 second | * |
|  |  | Not more than 20 second |  |
|  |  | Not more than 5 second |  |
| 24 | Pulse should be re-checked on every | 3 minutes |  |
|  |  | 2 minutes |  |
|  |  | 1 minutes |  |
| **Section D attitude of BLS** | | | |
| 25 | Self-assessment of reasons for the lack of BLS knowledge | Non-availability of professional training |  |
|  |  | Lack of interest |  |
|  |  | Busy curriculum |  |
|  |  | Combination of above three factors |  |
| 26 | Self-grading of BLS knowledge level | Poor |  |
|  |  | Below average |  |
|  |  | Good |  |
|  |  | Excellent |  |
