# Supplementary material for: Awareness, knowledge, and attitude towards basic life support among healthcare professional students in Bangladesh
Source: PLOS Glob Public Health. 2025 Sep 2;5(9):e0005126. doi: 10.1371/journal.pgph.0005126 (PMC12404403; doi:10.1371/journal.pgph.0005126)
Supplement: Attachment — Submitted filename: Response to Reviewers.docx [file pgph.0005126.s004.docx]

**Reviewer #1**

General comment -Need to work on English language. Some suggestions in language:

**Reviewer’s comment:** Abstract - results - "The current study revealed...lacking knowledge". This sentence needs grammar correction.

**Reply to the reviewer’s comment:** We appreciate your insightful recommendation, which helps in enhancing the manuscript's quality. In accordance with your advice, we have now amended it in the revised text.

**Reviewer’s comment:** Introduction - Line 7-12 appears to be out of context and does not gel with the whole paragraph. This needs to be re written.

**Reply to the reviewer’s comment:** Thank you for your comment. Corrected in the revised text.

**Reviewer’s comment:** Data collection procedures - Line 3 - "A feminisation data collection session". I don't understand what feminization means here.

**Reply to the reviewer’s comment:** Thank you for highlighting our unintentional mistake. The sentence has been updated in the revised text.

**Reviewer’s comment:** Data analysis - Line 4 - "...such as gender, faculty.". What is meant by faculty?

**Reply to the reviewer’s comment:** Thank you for highlighting our unintentional mistake. This will be the department rather than the faculty, as amended in the revised version.

**Reviewer’s comment:** In multiple sentences in Results section, the sentence starts with a number. This needs to be corrected. It is better not to start a sentence with a number.

**Reply to the reviewer’s comment:** I appreciate your insightful comment. In accordance with your recommendation, we have corrected the sentences in the revised text.

**Reviewer’s comment:** Individual component knowledge of BLS - Line 6 - " Merely 20% ...". This sentence needs restructuring.

**Reply to the reviewer’s comment:** Corrected in the revised text.

**Reviewer’s comment:** Results from regression analysis- Line 5 - need to re organise the sentence to highlight the significance of this.

**Reply to the reviewer’s comment:** I appreciate your insightful comment. The sentence is now reframed in the revised text.

**Reviewer’s comment:** Discussion - line 27 - "Bangladeshi final year ...'lacings' basic life support..."

**Reply to the reviewer’s comment:** Corrected in the revised text.

**Reviewer’s comment:** Discussion - line 38 - "In the study…. have positive ". This line needs to be modified.

**Reply to the reviewer’s comment:** Modified in the revised text.

**Reviewer’s comment:** Conclusion- Last line needs to be modified.

**Reply to the reviewer’s comment:** Modified in the revised text.

**Reviewer’s comment:** References 1 and 22 are same, 12 and 19 are same

**Reply to the reviewer’s comment:** We appreciate your careful feedback and regret our inadvertent error. We have now upgraded the reference list in the revised text.

Technical aspects: A. Questionnaire

**Reviewer’s comment:** 1. What is the significance of gender, height, weight and BMI in this study?

**Reply to the reviewer’s comment:** Thank you for your valuable question. This study includes demographic questions about the participants, such as age, gender, height, weight, and BMI, which are essential aspects of human research. However, as per your concern we removed the height, weight and BMI from the revised manuscript.

**Reviewer’s comment:** 2. Kindly provide the answers of question 18 and 19 with reference

**Reply to the reviewer’s comment:** Please refer reference 1 for the answer to the question 18 and 2 for 19.

1. Panchal, A. R., Bartos, J. A., Cabañas, J. G., Donnino, M. W., Drennan, I. R., Hirsch, K. G., Kudenchuk, P. J., Kurz, M. C., Lavonas, E. J., Morley, P. T., O’Neil, B. J., Peberdy, M. A., Rittenberger, J. C., Rodriguez, A. J., Sawyer, K. N., Berg, K. M., Arafeh, J., Benoit, J. L., Chase, M., … Magid, D. J. (2020). Part 3: Adult Basic and Advanced Life Support: 2020 American Heart Association Guidelines for Cardiopulmonary Resuscitation and Emergency Cardiovascular Care. *Circulation*, *142*(16_suppl_2), S366–S468. <https://doi.org/10.1161/CIR.0000000000000916>
2. Kleinman, M. E., Brennan, E. E., Goldberger, Z. D., Swor, R. A., Terry, M., Bobrow, B. J., Gazmuri, R. J., Travers, A. H., & Rea, T. (2015). Part 5: Adult Basic Life Support and Cardiopulmonary Resuscitation Quality. *Circulation*, *132*(18_suppl_2), S414–S435. https://doi.org/10.1161/CIR.0000000000000259

**Reviewer’s comment:** 3. Depth of CC - The total response is less than 486. Also, the result is hard to accept given the fact that most of the answers on basic questions like CPR rate and frequency were answered incorrectly while depth of CPR mostly had correct responses. The doubt further intensifies looking at the confusing options provided for the question

**Reply to the reviewer’s comment:** We appreciate you pointing out our mistake and posing this important question, which enhances the manuscript's quality. Regarding your issue, we double-checked the table and discovered that we had written 157 in place of 175; this has now been fixed in the updated text. We also acknowledged the concern that the question's unclear choice may have contributed to the majority of the right replies. We now acknowledge it in the limitation section.

**Reviewer’s comment:** B. BLS knowledge and ALS training correlation is not found because of the highly skewed value. This need to be acknowledged.

**Reply to the reviewer’s comment:** We now acknowledge it in the limitation section.

**Reviewer’s comment:** C. Limitations - This is a cross sectional survey. No skill was assessed in the study. This limitation needs to be acknowledged. Also, there is a possibility of bias inherent to all studies based on survey. Also, I was unable to understand the method of survey. Was it individually completed or as a group? Were they allowed to discuss or refer to any materials while completing the questionnaire?

**Reply to the reviewer’s comment:** Thank you for raising this issue. We now acknowledge the limitation in the revised text. We conducted the survey using the face-to-face interview method, which prohibited them from discussing this issue.

**Reviewer’s comment:** D. How was the scores of different components decided? One component/ question of the questionnaire might carry higher weightage compared to another one. Was this considered during scoring?

**Reply to the reviewer’s comment:** I appreciate your attention to this important issue. Initially, questions were not assigned a high value relative to other items in the scoring system; rather, one point was awarded for each accurate response. Thus, this issue has been included as a limitation in the limitations section.

**Reviewer #2**

The authors address an important subject (the knowledge of Basic Cardiac Life Support [BCLS] among final year students in 3 relevant health professions) in Bangladesh and the authors are to be complimented for addressing it.

Abstract

**Reviewer’s comment:** It would be important to note that three professions (medicine physio and nursing) are being examined. This is clear later in the text, but that is not early enough

**Reply to the reviewer’s comment:** Thank you for your comment. As per your recommendation we now upgraded the statement as follow: ‘’multi-center cross-sectional study was conducted across eight educational institutions in Dhaka, Jashore, and Sylhet division of Bangladesh from January to June 2024 which includes final year medicine, physiotherapy and nursing students.’’

Introduction

**Reviewer’s comment:** A brief description of the three programs is in order. Are these post-graduate or undergraduate courses, how many years of study, etc.? A small table comparing the three professions would be of use to the reader.

**Reply to the reviewer’s comment:** I appreciate your feedback. It is already covered in the manuscript's section on research design and participants. Only graduate students in their last year were included. We did not compare the results among the knowledge of various professional students since our goal was not to compare the BLS knowledge of the various professional students.

**Reviewer’s comment:** I suggest you delete the last two lines of the introduction “We anticipate… for healthcare professionals." Such sentiments belong in the discussion or conclusion.

**Reply to the reviewer’s comment:** Thank you for your comment. Removed form the revised manuscript.

Study design

**Reviewer’s comment:** How were the eight study institutions chosen? How many such in Bangladesh? Do they represent (a more or less) representative sample of institutions teaching the three professions? Public versus private? size? etc. If websites are available for the institutions involved, they should be added. “MBBS “should be changed to "medicine" throughout.

**Reply to the reviewer’s comment:** We chosen the institute based on the convenience of the researcher across three different cities, which includes both public and private colleges: physiotherapy students from Bangladesh Health Professional Institute (private, https://www.bhpi.edu.bd/), Saic Institute of Medical Science and Technology (private, https://saicmedical.edu.bd/), Jashore University of Science and Technology (public, https://ptr.just.edu.bd/), and National Institute of Traumatology and Orthopedic Rehabilitation (public, https://nitor.gov.bd/); final-year nursing students from Surma Nursing College (private https://mountadora.com/surma-nursing-institute/), and Sylhet Nursing College (public, website not available ); final year MBBS students from Parkview Medical College (private, https://www.pmch.edu.bd/) and Sylhet MAG Osmani Medical College (Public, <https://magosmanimedical.com/>). The word MBBS in now replaced with ‘’medicine" in the revised text.

Data collection procedures

**Reviewer’s comment:** Please explain “feminisation data collection session. How many data collectors were there?

**Reply to the reviewer’s comment:** Thank you for highlighting our unintentional mistake. The sentence has been updated in the revised text. Eight trained data collectors with degrees in medicine or physiotherapy were recruited to gather data.

Data collection tools

**Reviewer’s comment:** It would be interesting for the reader to be able to read the questionnaires. These could be included in an appendix. With respect to sociodemographic information, it is unclear why weight, height and BMI are included as they seem irrelevant to the study question.

**Reply to the reviewer’s comment:** Thank you for your suggestion. We already included the questionnaire as a supplementary file with this submission. This study includes demographic questions about the participants, such as age, gender, height, weight, and BMI, which are essential aspects of human research. However, as per your concern we removed the height, weight and BMI from the revised manuscript.

Results

**Reviewer’s comment:** One certainly does not need two decimal points for most of the data presented. This implies a level or resolution irrelevant to the data.

**Reply to the reviewer’s comment:** I am not getting the point.

**Reviewer’s comment:** The tables take up a lot of space and you have adequately summarised much of the relevant data in the text. They could be shortened or deleted

**Reply to the reviewer’s comment:** Thank you for the thoughtful feedback. We included it in the table for better visualization for the reader.

**Reviewer’s comment:** You should add the total number of participants in the legends. Eg in
table 1 “… towards BLS among 486 participants’’

**Reply to the reviewer’s comment:** Thank you for your comment. Added in the revised text.

**Reviewer’s comment:** “figure one and two" are mentioned in the text but I cannot find them

**Reply to the reviewer’s comment:** Please refer to the paragraph in the text where Figures 1 and 2 have been cited: ''Among the study participants, 34.2% reported the reasons for the lack of BLS knowledge were due to non-availability of professional training, 14.2% due to lack of interest, 12.3% due to busy curriculum, and 39.1% due to all of the factors (Figure 1). 41.4% of participants graded their BLS knowledge as poor, 38.9% as below average, 16.5% as good, and only 3.3% as excellent (Figure 2).''

**Reviewer’s comment:** p 13 Results from regression analysis: Information in the text with respect to female students does not seem to be consistent with what is seen in the table; please clarify.

**Reply to the reviewer’s comment:** Thank you for your comment. We now corrected the statement in the revised text as follow: ‘’The basic component of basic life support was better understood by female students (AOR 1.12; 95% CI 0.72–1.74, p = 0.624); nevertheless, the individual component of BLS was poorly understood by them (AOR 0.56; 95% CI 0.33–0.93, p = 0.025).’’

Discussion

**Reviewer’s comment:** I suggest the first sentence reads: “A fundamental understanding of emergency care is an essential competency for all health professionals, especially for those practicing the three disciplines examined here. “

**Reply to the reviewer’s comment:** Modified in the revised text.

**Reviewer’s comment:** Page16 - in the middle of the text the word “nevertheless" seems extraneous.

**Reply to the reviewer’s comment:** Corrected in the revised text.

Strengths, limitations and future recommendations:

**Reviewer’s comment:** I suggest that the first sentence read “To the best of our knowledge, this research is the first… “. One never knows if there is something else in the literature that examined what you did.

**Reply to the reviewer’s comment:** Thank you for your suggestion. Upgraded in the revised text.

**Reviewer’s comment:** Tables and text: I would change the word “department “to “discipline “throughout

**Reply to the reviewer’s comment:** Corrected in the revised text
