# Supplementary material for: Awareness, knowledge, and attitude towards basic life support among healthcare professional students in Bangladesh
Source: PLOS Glob Public Health. 2025 Sep 2;5(9):e0005126. doi: 10.1371/journal.pgph.0005126 (PMC12404403; doi:10.1371/journal.pgph.0005126)
Supplement: Attachment — Submitted filename: Response_to_Reviewers_auresp_2.docx [file pgph.0005126.s005.docx]

**Reviewer #1**

**Reviewer’s comment:** There are still some grammatical mistakes which needs to be addressed.

**Reply to the reviewer’s comment:** We appreciate your insightful recommendation, which helps in enhancing the manuscript's quality. In accordance with your advice, we have now amended it in the revised text.
